# The spectrum of mutations in families with breast and ovarian cancer from the region of Saxony, Germany

**DOI:** 10.1186/1897-4287-13-S1-A2

**Published:** 2015-09-09

**Authors:** Michalina Tomys, Marzena Skrzypczak-Zielinska, Marzena Jasinowska, Andrzej Plawski, Ursula Froster

**Affiliations:** 1 Institute of Applied Human Genetics and Oncogenetics, Zwenkau, Germany; 2 Institute of Human Genetics, Polish Academy of Sciences, Poznan, Poland

## 

Breast cancer is the most commonly occurring cancer among women. The disease frequently attacks women at a young age. The German population has been greatly affected by breast cancer with approximately 74,500 females and 600 males being diagnosed with the disease in 2012 alone. In many instances the cause of breast cancer is unknown. Genetic factors that greatly contribute to breast cancer are mutations of both *BRCA1* and *BRCA2 *genes. These occur in 10% of patients. Little is known so far about the contribution of other genes to the occurrence of breast cancer.

Here we report the mutation spectrum in genes that increase the risk of familial breast cancer in patients seeking genetic counseling in our Institute. The patients come from the region of Saxony in Germany, close to the Polish and Czech borders. Altogether we performed mutation analysis in 325 patients: 17 males and 308 females; deriving from a total of 216 families with breast and ovarian carcinoma.

All individuals were screened for mutations in six breast cancer susceptibility genes: *BRCA1, BRCA2, CHEK2, PALB2, RAD51, *and* RAD51C*. In the case of negative results and special family history we also analyzed other genes: *p53, ATM, PTEN, NBS1, NOD2, STK11, *and* CDH1. BRCA 1/2* were analyzed by multiplex-ligation-dependent probe-amplification (MLPA), PCR, and direct sequencing (including intron/exon boundaries). Other genes were analyzed by PCR and direct sequencing including intron/exon boundaries.

We observed *BRCA1* gene mutations in 15% of families (10% pathogenic and 5% unclassified variants). Three mutations were novel and not described. Of the *BRCA2* gene mutations we identified in 35% of families (12% pathogenic and 23% unclassified variant), 5 were not described before. In three families we found large genomic rearrangements in *BRCA1/2* by MLPA method. The *PALB2* gene mutations occurred in 8% of our probants, and the* CHEK2* mutation were observed in 4% of families. In 10% of families sequence variants in others analyzed genes were noted.

The total number of pathogenic mutations in our group of patients is compatible with the result from the German Familial Breast Cancer Consortium reporting 30% of mutations in *BRCA1* and *BRCA2* of families with a family history of breast cancer. However the spectrum of mutations is somehow different with more mutations in *BRCA2* and *CHEK2* as well as *PALB2*. Particularly for *CHEK2* we found a higher rate of pathogenic mutation [1]. This suggests that a screening spectrum of genes for familial breast cancer needs to be adjusted to the regional specification.
